# Intraperitoneal injection of lithium chloride induces lateralized activation of the insular cortex in adult mice

**DOI:** 10.1186/s13041-021-00780-z

**Published:** 2021-04-19

**Authors:** Kai Qian, Jiaqi Liu, Yiqing Cao, Jing Yang, Shuang Qiu

**Affiliations:** 1 Department of Neurobiology, Department of Anesthesiology of Second Affiliated Hospital, Zhejiang University School of Medicine, Hangzhou, 310058 China; 2 NHC and CAMS Key Laboratory of Medical Neurobiology, MOE Frontier Science Center for Brain Research and Brain-Machine Integration, School of Brain Science and Brain Medicine, Zhejiang University, Hangzhou, 310058 China; 3 School of Life Sciences, Center for Life Sciences, IDG/McGovern Institute for Brain Research, Peking University, Beijing, 100871 China

**Keywords:** Insular cortex, Lateralization, Vagotomy, LiCl, Fos staining

## Abstract

Insular cortex is a critical brain region that participates in the interoceptive sensations. Here, we combined the iDISCO + method and Fos immunostaining to confirm that the middle part of the right-side, but not the left-side, insular cortex in adult male mice is activated by intraperitoneal injection of lithium chloride. Lateralized activation of the insular cortex is also observed in adult female mice, but not in young or aged male mice. Furthermore, asymmetrical activation of the insular cortex was completely blocked when both sides of the vagal nerve are transected, whereas intravenous injection of lithium chloride has no effect on the insular activation. Combined together, these results indicate that the insular cortex unilaterally responds to aversive visceral stimuli in an age-dependent way and this process depends on the vagal afferent pathways.

## Introduction

The insular cortex is a brain structure implicated in multiple functions ranging from bodily sensation to emotional and salience processing, autonomic and motor control, decision-making, empathy and self-awareness. Among them, interoception, the physiological sense of the conditions of the body, is the key function of the insular cortex [1, 2]. The insular cortex receives inputs from both the outer body (auditory, visual and somatosensory signals) and the inner body (gustatory and interoceptive signals) and makes reciprocal connections with limbic systems, which is integral to the awareness of the body’s state [1, 3–6].

Functional imaging evidence in humans has shown that the insular cortex is activated during a wide variety of bodily sensations, such as thirst, pain, itch, dyspnea, sexual arousal, and distension of the stomach [1, 3, 7–9]. Damage involving the insula disrupts addiction to cigarette smoking in patients, demonstrating a role for the insular cortex in the representation of conscious bodily urges [10]. Researches in rodents confirm that the insular cortex is involved in chronic pain [11, 12], feeding behavior [13], drug addiction [14, 15], cardiac arrhythmia [16], taste coding [17, 18] and taste aversive memory [19, 20]. Inactivation or damage of the insular cortex prevents the urge to seek amphetamine [14, 15], opiate [21] or alcohol [22], blunts the signs of malaise induced by acute lithium administration [13, 14] and impaired taste aversive learning [23, 24].

The insular cortex can be subdivided, based on their cytoarchitecture (granular, dysgranular, and agranular), neural connections and function, into several areas. The posterior part of the insular cortex is mainly associated with sensorimotor processes. The intermediate part of the granular region of the insular cortex receives thalamic input from the VPLpc, and is also defined as a primary interoceptive cortex [25, 26], while the anterior part of the agranular region of insular cortex heavily interconnects with other cortical and subcortical regions and is more involved in high-level cognitive processes and affective processes [27, 28].

Intraperitoneal injection of lithium chloride (LiCl) induces abdominal malaise in rodents [29–31] and our previous work using Fos-immunostaining has shown that the middle part of the right-side, but not the left-side, insular cortex in adult male mice is significantly activated during LiCl treatment [13]. In this study, we combined immunolabeling-enabled imaging of solvent-cleared organs (iDISCO+) with Fos staining and confirmed the unilateral activation of the insular cortex in response to visceral malaise in male mice. In addition, we observed LiCl treatment-induced the lateralized activation of the insular cortex in adult female mice, but not in young or aged male mice, and we demonstrated that the vagal afferent pathway was required for the activation of the insular cortex.

## Methods

### Mice

C57BL/6J mice were obtained from the Shanghai SLAC Laboratory Animal Co., Ltd. Both male and female mice at 3 weeks to 15 months of age were used for data collection. All mice were kept on an inverse 12-h light/12-h dark cycle (lights off at 11:00). Mice were provided with ad libitum access to standard chow and water, unless otherwise noted.

 All animal care and experimental procedures complied with all relevant ethical regulations, were strictly conduct in accordance with the Guidelines for the Care and Use of Laboratory Animals of Zhejiang University and were approved by the Institutional Animal Care and Use Committee at Zhejiang University.

### iDISCO + immunolabeling and 3D imaging

The protocol for iDISCO + was based on the reported technique [32, 33]. Briefly, adult mice were anesthesized with 1 % pentasorbital sodium and perfused with an intracardiac perfusion of 1*×* PBS followed by 1 % paraformaldehyde (PFA) in PBS. All harvested brain samples were post-fixed overnight at 4 °C in 1 % PFA in PBS. Brain samples were then processed by the iDISCO + immunolabeling and tissue optical clearing and imaged on the LaVision Biotec Ultramicroscope II.

### Vagotomy

Mice were anaesthetized with 1 % pentasorbital sodium and placed in a stereotaxic apparatus while resting on a heating pad. A midline abdominal incision from the xiphoid process (about 3-cm long) was made along the linea alba to expose the abdominal cavity, and the liver was gently retracted to the left side with cotton swabs. The stomach was then moved out of the cavity and kept moisturized with saline throughout the surgery. The oesophagus was gently lifted and all identifiable vagus nerve fibres above the hepatic branches of the anterior vagus (both anterior and posterior vagal trunk) were excised with micro-scissors to achieve total subdiaphragmatic vagotomy (Fig. 6a). Sham vagotomy consists of the same procedure without touching the esophagus or nerves. Vagotomy impairs gastric emptying, therefore, all experiments with vagotomized mice were completed within 6 h of the surgery, to avoid any confounding effects caused by the impaired gastrointestinal flow without additional pyloroplasty surgery. Furthermore, mice were provided with liquid food jelly instead of chow food for a day before vagotomy to minimize the amount of solid ingesta in the stomach during the experiment. At the time of perfusion, the stomachs of vagotomized mice were found to be severely expanded and filled with jelly, indicating impaired gastric motility resulting from the vagotomy.

### Immunohistochemistry

Mice were perfused with 1*×* PBS of room-temperature, followed by 4 % PFA in 1*×* PBS. Brains were removed and postfixed for 48 h at 4 °C. Coronal sections were cut with a vibratome at 40 *µ*m thickness. Sections were blocked for 2 h in blocking buffer (1 % normal goat serum and 0.3 % Triton X-100 in PBS) and incubated with rabbit anti-Fos antibody (SYSY system, 226003, 1:10,000 dilution, RRID:AB_2231974) at room temperature for 48 h. After three washes in 1× PBS, sections were incubated in Alexa Fluor 555 donkey anti-rabbit secondary antibody (1:2000 dilution; Thermo Scientific). Sections were mounted and imaged on an Olympus VS120 microscope with a 10× objective. For detection of Fos in mice with different aversive stimuli, mice were perfused 1.5 h after injection (i.p.) with different regents or electric foot shock unless otherwise mentioned.

### Data analysis

Quantification of Fos staining was performed on every 6th slice in the LHA from Bregma − 0.7 to − 1.92 mm (6 sections per mice) and on every third slice in the following area: PBN from Bregma − 5.02 to − 5.40 mm (3 sections per mice), NTS from Bregma − 6.96 to − 7.48 mm (5 sections per mice). We divided the insular cortex (IC) into three segments anterior insular cortex (aIC, from Bregma + 2.46 to + 1.34 mm), middle insular cortex (mIC, from Bregma + 1.34 to − 0.02 mm) and posterior insular cortex (pIC, from Bregma − 0.02 to − 1.46 mm), Quantification of Fos staining in the whole IC was performed on every third slice from Bregma + 2.46 to − 1.06 mm (28 sections per mice). All images were subsequently overlaid with the corresponding atlas section to anatomically define the regions of interest. Positive cells lying on the boundary were excluded. A cell was considered positive only if it displayed an intensity value above the intensity threshold of the background. Quantification was performed using the cell counter tool in ImageJ.

### Statistical significance

All data are presented as the mean ± SEM. P < 0.05 was considered statistically significant. Data were analyzed with the unpaired t-test or one-way ANOVA, using repeated measures where appropriate. Significant ANOVAs were followed by a post-hoc Turkey’s test where appropriate.

## Result

### Anatomical characterization of aversive visceral stimuli analyzed by iDISCO + method

**Fig. 1:**
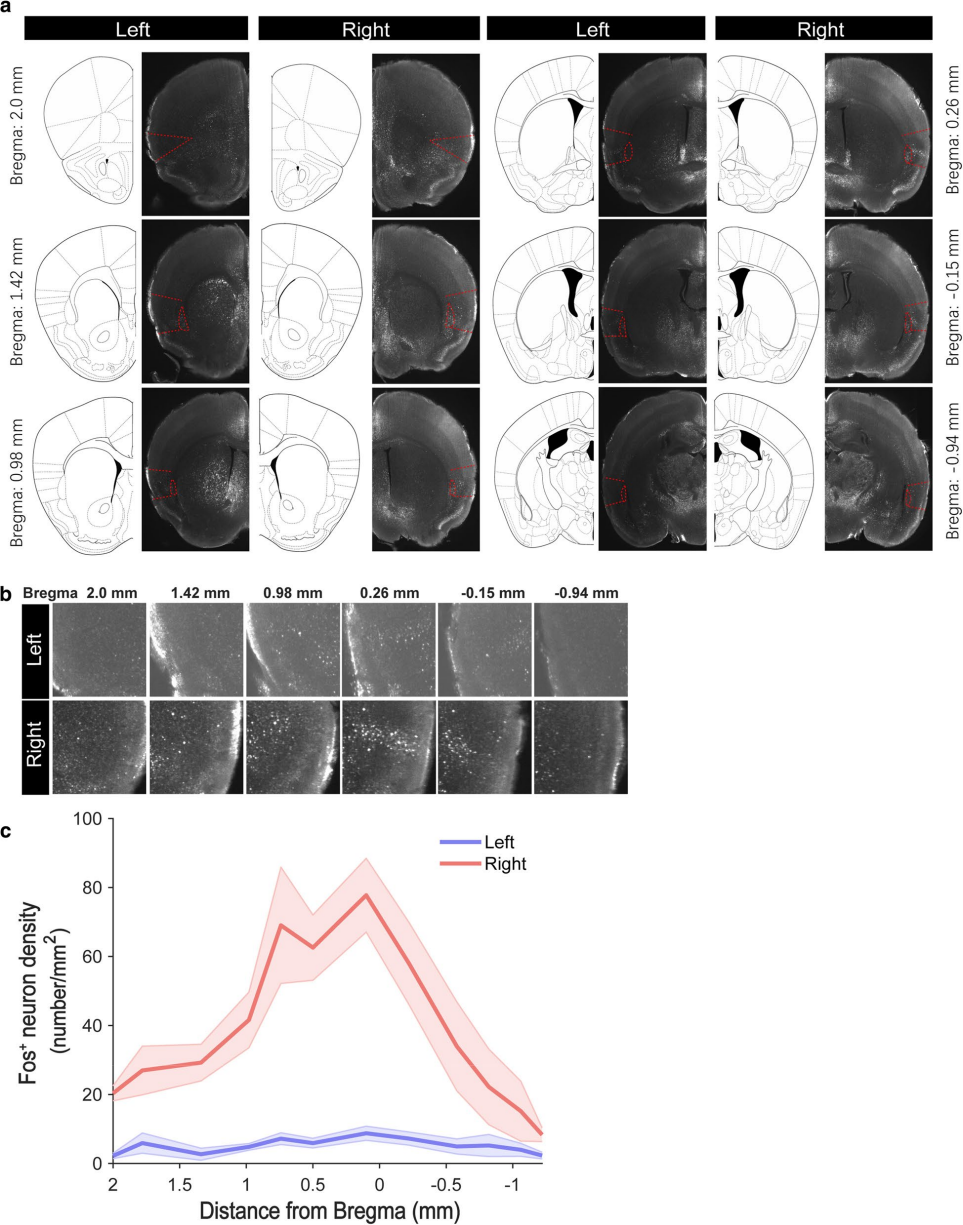
Whole-tissue Fos^+^ Immunolabeling of Adult Brains by iDISCO + method. **a** Expression of Fos-like immunoreactivity along the entire length of the anterior-posterior (A-P) axis of the insular cortex after intraperitoneal injection of LiCl (15 mg/kg). The left panel in each column was the reference annotation image while the right was the raw data of optical sections of lightsheet imaging. Totally, 6 representative segments of the insular cortex were selected and shown (Bregma 2.0 mm, 1.42 mm, 0.98 mm, 0.26 mm, − 0.15 mm, − 0.94 mm). **b** Panels are higher-magnification images of insular cortex along the A-P axis. **c** Scatter plot displaying the density of Fos^+^ immunolabeling neurons in the insular of adult mice (n = 6 mice per group, Data are presented as means ± SEM)

To characterize brain circuits engaged by anorexigenic signals, we used Fos, an intermediate-early gene with well-characterized activity-dependent expression [34], to identify anatomical structures in which neurons were activated following aversive delivery. We have observed that Fos expression after intraperitoneal (i.p.) injection of anorexigenic signals, such as lithium chloride (LiCl), which causes nausea and visceral malaise, induces a robust activation of Fos in the middle part of the right, but not the left insular cortex [13]. After the injection of LiCl, the mice showed barely no locomotion and crouched in the corner of the homecage with frequent abdominal spasms. About 1 h later, diarrhea would happen to the mice. To gain a better understanding of the landscape of Fos expression in the insular cortex and throughout the brain, we used the iDISCO + method, which is a pipeline for high-speed acquisition of brain activity at cellular resolution through profiling immediate early gene expression using whole-tissue immunolabeling and light-sheet 3D imaging, followed by automated mapping and analysis of activity by an open-source software program ClearMap. Results obtained from automated segmentation of the brain regions were confirmed by inspection of both voxelized density maps and raw data (Fig. 1a). Compared with reference annotation, we observed Fos^+^ neurons along the entire length of the anterior-posterior (A-P) axis of the insular cortex but with a greater number within the middle part of the insular cortex (Bregma between − 0.15 and 0.26 mm) (Fig. 1a) (*t*_12_ = 6.34, *P* < 0.0001). Using total cell counts and peak intensity counts, we extracted digital positions of segmented cells in the insular cortex and plotted their density along the A-P axis. The density of Fos^+^ neuron was significantly higher in the right side of the insular cortex relative to the left side (Fig. 1b, c), especially in the Bregma of 0.1 mm. Together, our iDISCO + imaging data indicate that aversive visceral stimuli induces a robust neuronal activation in the middle part of the right insular cortex in vivo.

### Lateralized activation of the insular cortex in adult female mice

**Fig. 2:**
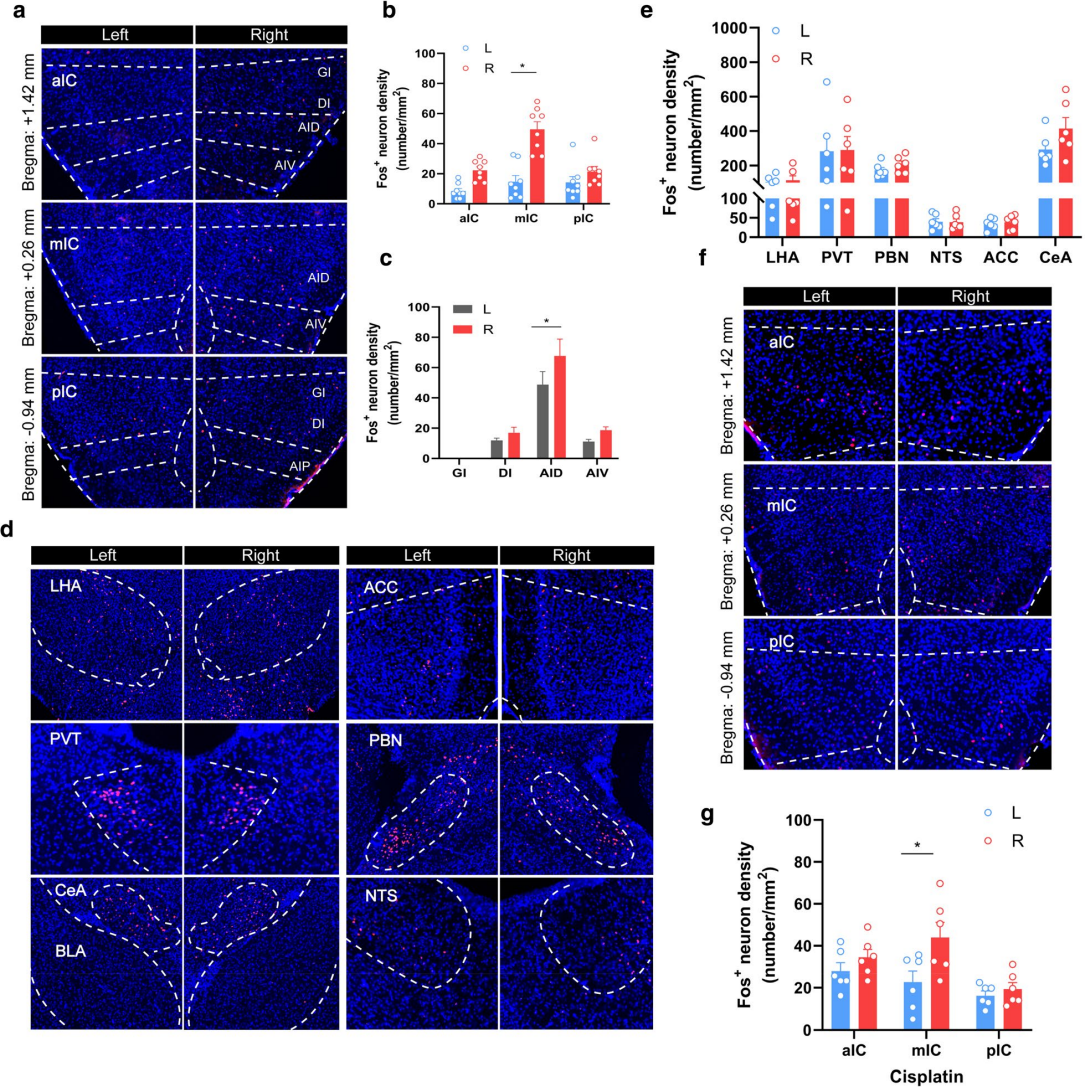
Activation of different brain regions by abdominal aversive stimuli in female mice. **a**, **b** The insular cortex was divided into three segments aIC, mIC and pIC. Representative histology (**a**) and quantification (**b**) of Fos-like immunoreactivity in the left- and right-side along the entire length of A-P axis of the insular cortex after intraperitoneal injection of LiCl (150 mg/kg). **c** Quantification of Fos-like immunoreactivity in AID, DI, GI, AIV. **d**, **e** Representative histology (**d**) and quantification (**e**) of Fos-like immunoreactivity in the left and right PVT, ACC, LHA, PBN, CeA, NTS after intraperitoneal injection of LiCl (150 mg/kg). **f**, **g** Representative histology (**f**) and quantification (**g**) of Fos-like immunoreactivity in the left and right insular cortex after intraperitoneal injection of Cisplatin (4 mg/kg). *IC* insular cortex, *aIC* anterior insular cortex, *mIC* middle insular cortex, *pIC* posterior insular cortex, *PBN* parabrachial nucleus, *CeA* central amygdala, *ACC* anterior cingulate cortex, *NTS* nucleus of solitary tract, *LHA*, lateral hypothalamus, *PVT* paraventricular thalamic nucleus, *AID* agranular insular cortex, dorsal part; *DI* dysgranular insular cortex, *GI* granular insular cortex, *AIV* agranular insular cortex, ventral part. n = 8 mice per group. Data were presented as means ± SEM. Turkey’s test analysis between each group as indicated *P < 0.05. Scale bar, 100 μm

Since the adult male mice were used in the previous experiment, we next examined whether similar phenomenon are observed in the female mice. We therefore injected (i.p.) LiCl into the adult female mice and found that LiCl induced Fos positive (Fos^+^) neurons were mainly localized within the segment between 1.26 mm before Bregma (Bregma + 1.26) and 0.02 mm after Bregma (Bregma-0.02) of the right insular cortex (Fig. 2a, b) (mIC, *t*_14_ = 2.186, *P* = 0.047). Notably, the activated areas were concentrated in the agranular insular cortex, dorsal part (AID) (AID, *t*_25_ = 2.297, *P* = 0.030) and dysgranular insular cortex (DI), but not in granular insular cortex (GI) (Fig. 2c). Visceral afferents ascending are topographically organized in the granular and dysgranular fields of the insular cortex, whereas the agranular cortex appears to receive highly integrated limbic afferents from the infralimbic cortex and the mediodorsal nucleus of the thalamus [25]. Additionally, LiCl induced substantial Fos expression bilaterally in several brain regions, including the central amygdala (CeA), the anterior cingulate cortex (ACC), the parabrachial nucleus (PBN), the nucleus of solitary tract (NTS), the lateral hypothalamus (LHA), and the paraventricular thalamic nucleus (PVT) (Fig. 2d, e), which have been reported to be involved in pain or interoception [13, 35–38]. Similarly, when Cisplatin was injected intraperitoneally, the activation of the right-side insular cortex was also observed (Fig. 2f, g). These results indicate that the right-side middle part of the insular cortex of the female mice responds to aversive visceral stimuli, in consistence to the male mice.

### No obvious LiCl-induced insular activation in the young or aged male mice

**Fig. 3:**
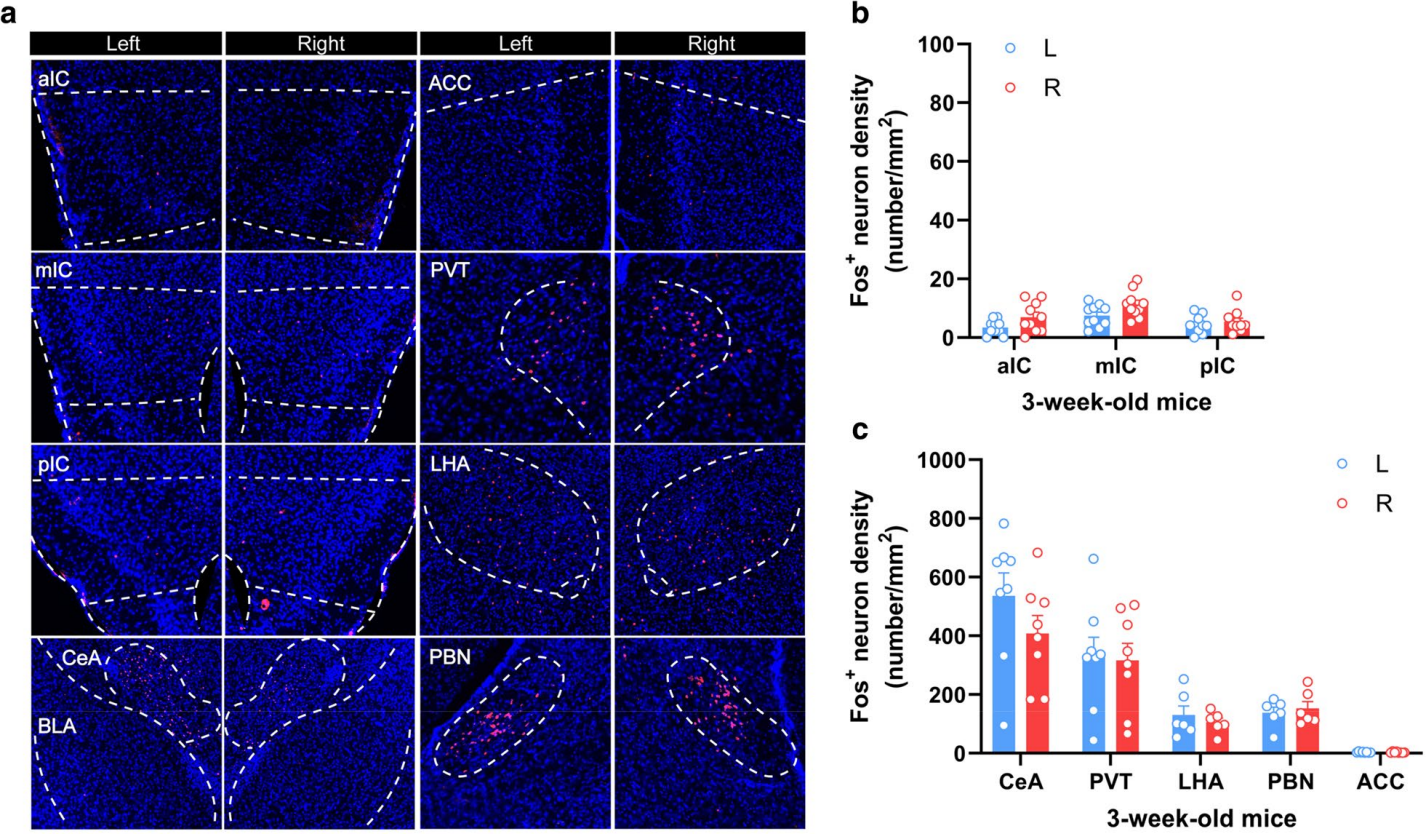
Fos^+^ immunostaining in different brain regions of the young male mice induced by i.p. injection of LiCl. **a–c** Representative histology (**a**) and quantification (**b**, **c**) of Fos-like immunoreactivity in the left- and right-side of the aIC, the mIC, the pIC, the CeA, the PVT, the LHA, the PBN, and the ACC after intraperitoneal injection of LiCl (150 mg/kg). CeA, central amygdala. n = 6–8 mice per group, Data were presented as means ± SEM. Scale bar, 100 μm

**Fig. 4:**
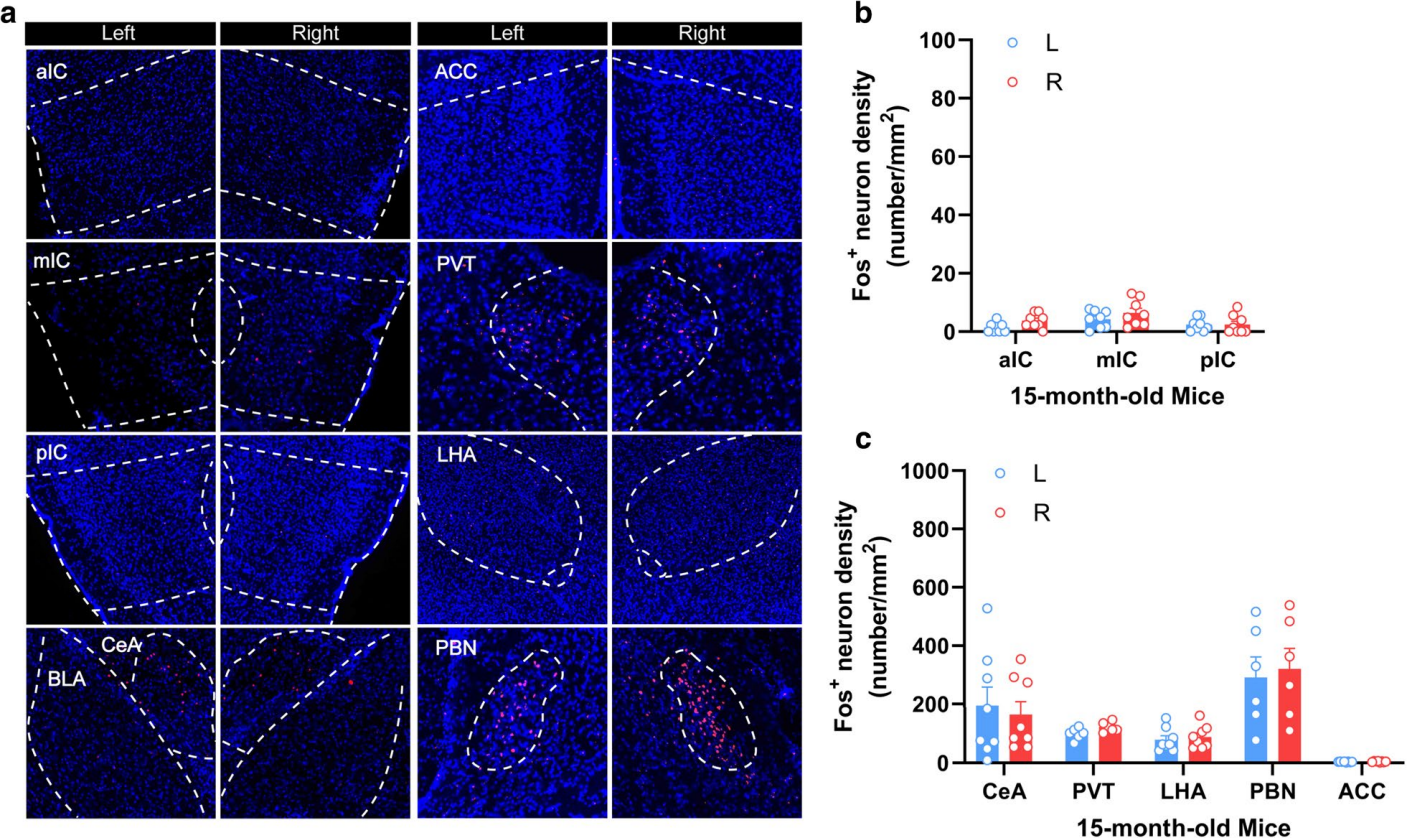
Fos expression in different brain regions of the aged male mice induced by i.p. injection of LiCl. **c** Representative histology (**a**) and quantification (**b**, **c**) of Fos-like immunoreactivity in the left- and right-side of the aIC, the mIC, the pIC, the CeA, the PVT, the LHA, the PBN, and the ACC after intraperitoneal injection of LiCl (150 mg/kg). n = 6–8 mice per group. Data were presented as means ± SEM. Scale bar, 100 μm

Previous studies in rodents have shown that adolescents exhibit differences from adult rodents on measures of fear-, anxiety- and depression-related behaviors and reactivity to stress [35, 36], and alteration of CREB phosphorylation and spatial memory deficits in aged mice [37]. It’s necessary to assess whether and to what extent the aversive visceral stimuli-induced insular activation is age-dependent. Our immunohistochemical data revealed that LiCl induced substantial Fos^+^ expression bilaterally in several brain regions, such as the central amygdala (CeA), the PVT, the LHA, and the PBN in 3 week-old mice. The density of Fos^+^ neurons in the CeA and the PVT were obviously higher than that in the LHA and the PBN (Fig. 3a, c). However, the whole insular cortex, as well as the ACC, was rarely activated (Fig. 3a, b). We next tested the aged mice (15 months, 15 M) using similar protocol. As illustrated in Fig. 4a, I.P. injection of LiCl induced no obvious neuronal activation in the whole insular cortex of the 15 M male mice (Fig. 4a, b). An age-related reduction of Fos^+^ neurons expression also occurred in the CeA, the PVT, and the LHA, but not in the ACC (Fig. 4a, c). Taken together, these results indicate that neuronal activation of the insular cortex induced by aversive visceral stimuli is age-dependent, and obvious activation is only observed in the insular cortex of the adult mice, but not in that of the young and aged mice.

### Intravenous injection of LiCl induced no obvious Fos expression in the insular cortex

**Fig. 5:**
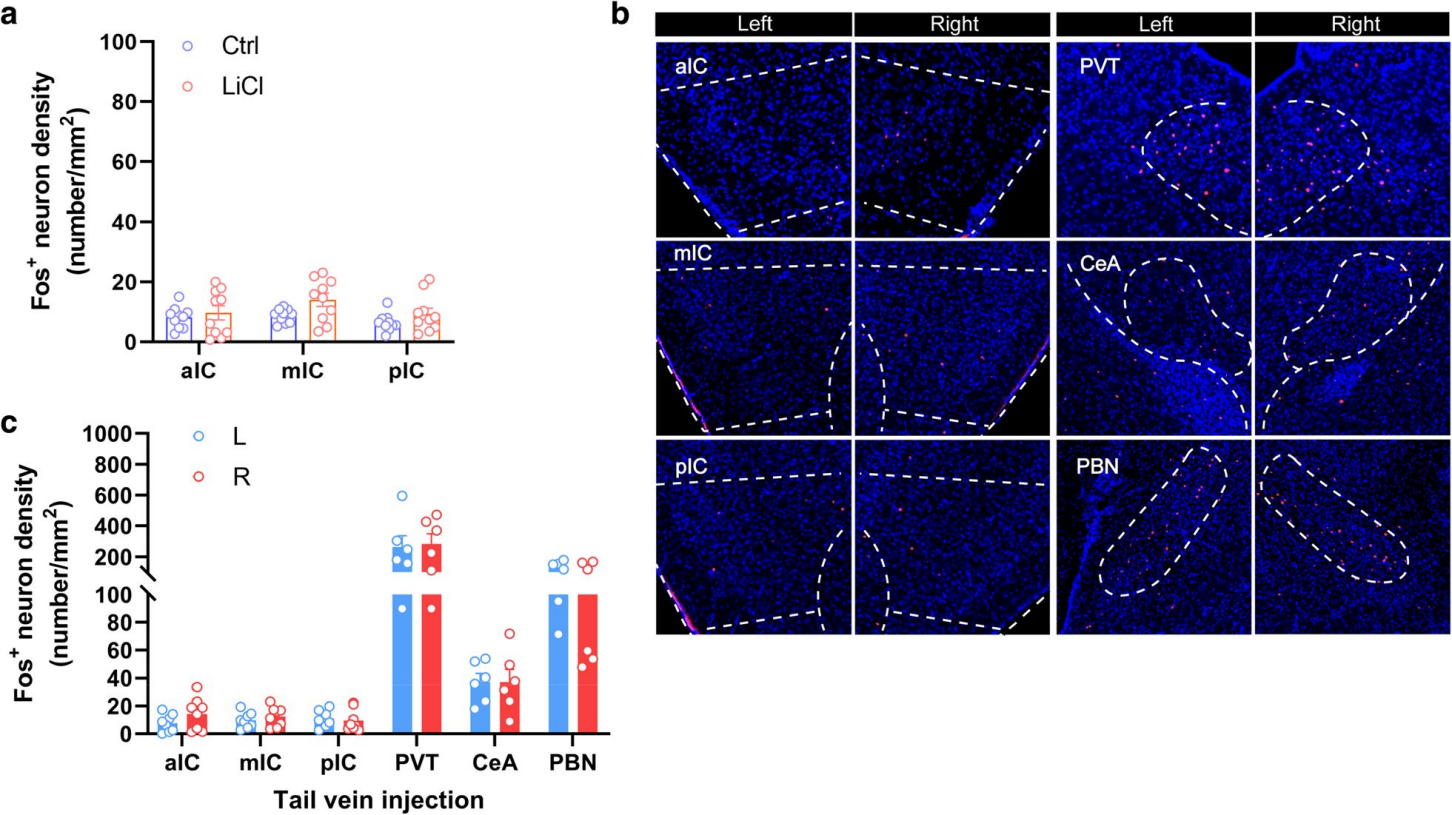
Tail vein injection of LiCl induces no obvious Fos expression. **a** Quantification of Fos-like immunoreactivity in the aIC, the mIC, and the pIC after intraperitoneal injection of Saline or LiCl (15 mg/kg) (n = 10 mice per group, F (2, 57) = 1.694, *P* = 0.193, Data are presented as means ± SEM). **b**, **c** Representative histology (**b**) and quantification (**c**) of Fos-like immunoreactivity in the left- and right-side of the aIC, the mIC, the pIC, the PVT, the CeA, and the PBN. Data were presented as means ± SEM, Scale bar, 100 μm

To identify the possible pathways involved in the LiCl-induced insular activation, we first focused on the blood circulatory system. Lithium has the anti-convulsant effects both in human and in rodents [38, 39], and the intravenous route of delivery is the most efficient means of delivering substances to animals because it bypasses the need for solute absorption [40]. Here, we intravenously injected LiCl (15 mg/kg) via tail vein, and observed that intravenous administration of LiCl resulted in a specific distribution of Fos^+^ activity throughout the mice brains, such as dense labeling was observed throughout the PVT, moderate numbers of Fos^+^ neurons were observed in the PBN, whereas, scattered labeled cells were observed within CeA. It’s noteworthy that extremely few Fos^+^ neurons were observed in the insular cortex from anterior to posterior (Fig. 5b, c). These data suggest that lateralized activation of the insular cortex is not due to the LiCl in the circulatory system.

### The effect of subdiaphragmatic vagotomy on fos expression

**Fig. 6:**
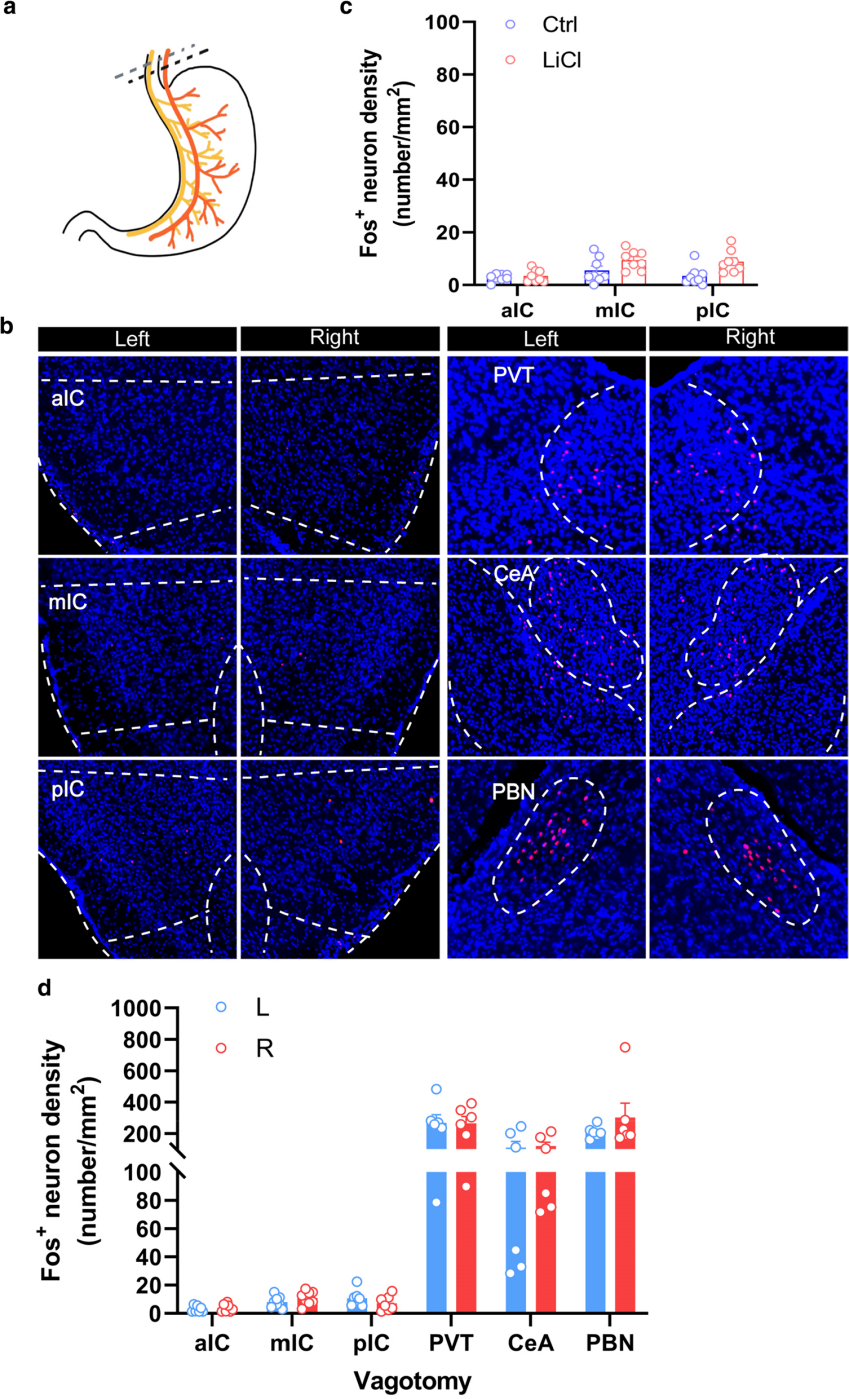
Subdiaphragmatic Vagotomy impairs LiCl-induced Fos^+^ Expression in the insular cortex **a** Bilateral branches of the both anterior and posterior vagal trunk were excised with micro-scissors to achieve total subdiaphragmatic vagotomy. **b** Representative histology of Fos-like immunoreactivity in the left- and right-side of the aIC, the mIC, the pIC, the PVT, the CeA, and the PBN after intraperitoneal injection of LiCl (150 mg/kg). **c**, **d** Quantification of Fos-like immunoreactivity in the upper regions [n = 10 mice per group, F (2, 42) = 2.072, *P* = 0.139] after intraperitoneal injection of LiCl (150 mg/kg). Data were presented as means ± SEM. Scale bar, 100 μm

We next asked whether vagal nerve afferent pathway is necessary for the activation of the insular cortex in response to i.p. injection of LiCl. We detected the number of the Fos^+^ neurons in the insular cortex of the mice with or without subdiaphragmatic vagotomy (Fig. 6a) and observed no obvious LiCl-induced activation of the insular cortex in the mice with subdiaphragmatic vagotamy compared with the sham group (Fig. 6b, c). However, we still observed Fos^+^ neurons in some brain regions such as the PVT, the CeA, and PBN (Fig. 6b, d). These data indicate that the vagal afferent pathway is required for the activation of the insular cortex induced by aversive visceral malaise.

## Discussion

In this study, we demonstrate that the insular cortex is asymmetrically activated in response to aversive stimuli. Moreover, this process is gender-independent and age-dependent. We also found that the vagal afferent pathway is necessary for the insular activation induced by abdominal malaise.

The insular cortex is a bilaterally located brain region in both rodents and humans. Bilateral brain regions are often assumed to have the same function. However, human imaging studies have shown that the insular cortex is more activated on one side under particular conditions [1, 3, 7], indicating that lateralization of the insular cortex may be the norm rather than an exception. For example, greater activation of the left insular cortex is associated with both maternal and romantic love [41, 42], seeing or making a smile [43], hearing happy voices or pleasant music [44, 45], while activation of the right insular cortex is associated with recall-generated sadness or anger [46], anticipatory anxiety and pain [47], panic, sexual arousal [48] and disgust [49]. Lateralized activation of the insular cortex is also observed in rats [50]. In this study, together with our previous finding [13], we certificate that the mouse insular cortex functions asymmetrically and the right-side insular cortex responds to aversive visceral stimuli, in consistent with the findings in humans that the right insular cortex is specialized to process negative emotions [51]. Interestingly, we observed similar phenomena in adult female mice, indicating that the insular lateralization is not sex specific. However, the insular activation was not detected in young mice or in aged mice, suggesting that the function of the insular cortex in response to external or internal stimuli is developmentally influenced.

Here, to identify the possible pathways involved in the LiCl-induced insular activation, we firstly exclude the possibility that LiCl treatment exerts effect on the insular cortex directly through circulatory system. We injected LiCl intravenously and did not observe the activation in the insular cortex. By now, there are two nervous afferent pathways that have been identified to participate in the crosstalk between the gut and the brain, one is via vagal nerve and the other is via spinal nerve. We next transected vagus nerve bilaterally at the supradiaphragmatic level and found that vagotomy completely blocks the activation of the insular cortex, indicating that vagal nerve afferent pathway is necessary for the activation of the insular cortex in response to visceral stimuli [1, 52]. Interestingly, one research has shown that the vagus nerve functions asymmetrically in stimulating brain reward neurons [53]. We cannot exclude the possibility that the vagal afferent pathway also contributes to the lateralized activation of the insular cortex. It is important to further decipher the molecular, neuronal and circuitry mechanism underlying the lateralization of the insular cortex in the future.

Recently, more and more researches have shown that the rodent brain functions in an asymmetric way, such as ACC in empathy [54], Amygdala in pain [55], hippocampus in spatial memory and navigation [56], and auditory cortex in social communication [57]. Here, we demonstrate that the insular cortex functions laterally in sensing aversive stimuli. These findings indicate that brain lateralization in rodents may provide for fine-tuning the immediate response to injury and how the response changes over time, just like the functional lateralization in human language areas providing for specialization over redundancy. It also provides possibility that researchers may utilize rodent models to find out the mechanisms underlying brain lateralization.

## Data Availability

Please contact author for data requests.
